# Current Feeding Practice of *Xenopus laevis* in a Laboratory Setting

**DOI:** 10.3390/ani12091163

**Published:** 2022-05-01

**Authors:** Linda F. Böswald, Dana Matzek, Bastian Popper

**Affiliations:** 1 Chair for Animal Nutrition and Dietetics, Faculty of Veterinary Medicine, Ludwig-Maximilians-Universität München, Schönleutnerstr. 8, 85764 Oberschleißheim, Germany; linda.boeswald@lmu.de; 2 Biomedical Center, Core Facility Animal Models, Faculty of Medicine, Ludwig-Maximilians-Universität München, Großhaderner Straße 9, 82152 München, Germany; dana.matzek@med.uni-muenchen.de

**Keywords:** amphibian, model organism, African clawed frog, nutrition, survey

## Abstract

**Simple Summary:**

The standardization of husbandry conditions, feeding regimens, and diets is the prerequisite for the comparability of results generated by the use of laboratory animals. Compared to rodents, which account for the largest proportion of laboratory animals worldwide, standardization is still inadequate for aquatic species, especially clawed frogs (*Xenopus laevis*). In this context, species-specific feeding is important for standardization and animal health and welfare. However, the current feeding recommendations for *Xenopus* are extrapolated from other species’ nutrient requirements. In addition, the feeding regimen itself affects animal behavior. In particular, the feed intake seems to be influenced by the type of feed. Using a questionnaire, the most common methods of husbandry and feeding of *Xenopus laevis* in laboratory settings were recorded, and the feeds were compared to the recommendations by Ferrie. The results showed variations with regard to husbandry and feeding concepts between facilities. Commercial Xenopus diets and fish feed were the most commonly used feeds, all meeting the recommendation for protein content but differing considerably in mineral content (based on labelled information). It appears that the feed composition and feeding regimen need to be the focus of further research to ensure that feeding and husbandry are adapted to nutritional and behavioral needs.

**Abstract:**

African clawed frogs are common animal models used in various research areas. However, husbandry and especially feeding regimens are not nearly as standardized as is established for other laboratory animals. We recorded the diets and feeding protocols commonly used in laboratory practice in a questionnaire (18 responses). The survey revealed a wide variety of housing conditions. Feeding protocols and, in particular, diet composition varied considerably between facilities. While diets tailored to Xenopus were used in the majority, differences in feeding frequency and dietary components were noted. From five responses, the weekly feed intake per frog could be calculated, showing considerable differences in dry matter intake (1.37–5.4 g). The labelled nutrient content of the diets fed in the facilities (n = 10) met the recommendations in most cases, with protein as the major energy source. However, the mineral content varied markedly between diets. Both floating and sinking diets were used, while quickly sinking diets were associated with feed leftovers. Feed processing may likely influence feed intake behavior. Further research is needed to ensure standardization for aquatic species with respect to husbandry systems, feeding regimens, and especially the nutrient composition of feeds. Furthermore, this work will contribute positively to animal welfare and the comparability of research results.

## 1. Introduction

The Xenopus frogs (mainly *Xenopus laevis*, the African Clawed Frog, and *Xenopus tropicalis*, the Tropical Clawed Frog) have been used as research animals for almost a century. Today, the main fields of research with Xenopus species are embryology, molecular biology, genetics, immunology, and toxicology [1,2,3,4,5,6].

Xenopus frogs are aquatic amphibians in the family Pipidae. Compared to many mammals, they are relatively easy to keep in a laboratory setting, while having a short generation interval and a long lifespan of 15 to even 30 years [2,4]. The oocytes can be accessed directly because they are outside of the female’s body. This makes experiments that would be hard to conduct in mammals due to ethical and practical reasons possible [3]. Especially after hormonal stimulation, a high quantity of oocytes can be obtained [7]. In addition, there are now several genetically modified strains available [3]. These factors make Xenopus frogs an attractive model organism for research.

In their natural habitat, the carnivorous Xenopus frogs are adapted to feeding on prey animals. They adopt a motionless waiting position in the water until they sense the appearance of prey, relying on visual, olfactory, and tactile stimuli as well as vibration. If prey is in the vicinity of the frog, it will start a “scooping” behavior with its forelimbs, pushing the prey into its oral cavity, shoving it inside its mouth with the back of the hands [8,9]. The feeding behavior can be described as rather unselective [8]. However, the recognition of feed items seems to be linked to their location, with a preference for items sinking through the water. Based on the literature, feed items lying on the bottom of the tank are not consumed [4,10].

There is no specific data on the nutrient requirement of Xenopus frogs. The recommendations for nutrient intake that exist in the literature are based on an extrapolation of nutrient requirements of other species such as fish, poultry, rats, dogs, and cats [4,11,12]. While this is the method of choice for an orientation about adequate nutrient supply for a species that has not been investigated further, it leaves a high level of uncertainty. In laboratory practice, many different feeding regimens for Xenopus frogs exist [13].

Besides the standardization of feeding regimens for the comparability of studies, the composition of the feed influences the experimental animals [14] and thus the results. The standardization of nutrition in animal studies is required for aquatic species such as clawed frogs.

In the present study, we aim to get an overview of Xenopus frog feed and feeding regimens in experimental facilities.

## 2. Materials and Methods

### 2.1. Questionnaire

A questionnaire was set up via the online tool SurveyMonkey™ (Momentive Europe UC, Dublin, Ireland). The questionnaire was presented in German with the English translation of each question and answer directly below the German text. It contained 28 questions (Supplementary Table S1).

The link to the online questionnaire was distributed to colleagues directly, encouraging them to share the link themselves, and via a mailing list of the Munich university network addressing animal facility heads, animal welfare officials, and members of the authorities. The facilities participating in the survey were spread over several countries (Supplementary Table S2). It was open from 20 October 2021 to 10 January 2022.

### 2.2. Calculations

The labelled nutrient content of the commercial diets named in the questionnaire was obtained through the manufacturers´ datasheets. If an amount of feed was given in grams and could be connected to the number of animals in the tank and per week, a ration calculation was conducted (energy and nutrient supply per animal per week). The energy and nutrient content of the diet, consisting of either one single feed or several feeds combined in given percentages, was expressed on a dry matter (DM) basis and compared with the current recommendations for Xenopus frogs [11].

## 3. Results

### 3.1. Questionnaire

There were 20 replies to the questionnaire. However, there were three responses by one facility, which we had to correct: we chose to use the response given by a veterinarian (presumably the head of animal husbandry) and eliminate the two answers by non-veterinarian researchers. Thus, 18 complete replies were used for the further evaluation.

#### 3.1.1. General information

The majority of respondents (50.0%) are researchers (not veterinary), followed by animal technicians (22.2%). Nearly all responses come from university facilities (94.4%), with only one response from a biotechnology company. Most facilities have a long experience of keeping Xenopus frogs (>15 years: 66.7%; 11–15 years: 5.6%) and keep more than a hundred individuals (101–300 frogs: 38.9%; >300 frogs: 38.9%).

The frogs are mostly used for different research purposes: spawning for reproductive medicine and developmental biology (22.2%), spawning for research other than reproductive medicine (77.8%), organ removal (33.3%), toxicity/substance testing (5.6%), behavioral research (5.6%), or other purposes (27.8%; e.g., microbiology, immunology, nutrition and metabolism research, education, and breeding).

#### 3.1.2. Housing

The housing consists of plastic tanks with automated water circulation in more than three quarters of the facilities (removable tanks: 55.6%; permanently installed tanks: 22.2%). In two facilities, fiberglass or plastic tanks are in use, respectively. The groups of frogs consist of 6–15 animals in 66.7% of the facilities. The frogs are kept at a water temperature of 19–20 °C (50.0%) or <18 °C (44.4%), with only one facility reporting 21–22 °C and none reporting temperatures higher than 22 °C. The water level in the tanks was at a minimum of 14 cm (14–20 cm: 50.0%; 21–30 cm: 27.8%). Several facilities reported quite deep tanks with a water level of 41–50 cm (11.1%) and >50 cm (11.1%).

Some of the facilities do not offer any enrichment (22.2%), while most have plastic shelters or houses available in the tanks (55.6%). Natural material such as live plants or mangrove roots is not used at all. Other enrichment items in use are plastic or clay tubes or shelters made from half clay flower pots. The light cycle is typically set for 12 h of light followed by 12 h of dark (83.3%), in all cases determined by artificial light sources. The median reported light intensity reaching the tank surface was 175 lux (range 50–400; the mean was 203).

#### 3.1.3. Feeding

Respondents estimate the nutritional status of their Xenopus frogs to be rather well nourished (66.7%) or normal (33.3%). There is a broad range of feeding frequencies reported: 50.0% feed three times a week, 22.2% feed two times a week, 5.6% feed once a week, 5.6% feed less than once per week, and 16.7% have different feeding intervals (2 facilities daily Monday to Friday, one facility twice daily Monday to Thursday). The most commonly used feed (multiple choice question) is a commercial feed for *Xenopus laevis* (55.6%), followed by commercial fish feed (50.0%), but one facility also uses live feed animals. The diets are mostly pelleted (83.3%) or pelleted extrudate (16.7%). Movement of the diet in water varies quite a bit: 44.4% reported it as floating, 33.3% as sinking to the ground quickly, and 22.2% as sinking to the ground slowly.

Most facilities do not administer supplementary feed in addition to the regular diet (88.9%). One facility seldom feeds live earthworms and one facility supplements raw liver cut into small pieces if the egg quality declines.

The amount of feed per tank and meal is assigned based on the animal technicians´ experience (61.1%) or according to the frogs´ behavior after the meal (38.9%), i.e., whether they resume a resting position (seemingly satisfied) or remain active (still “hungry”). Only in two facilities (11.1%) is the weight development of the animals used to adjust the amount of feed. The amount of feed is defined in gram (50.0%), number of pellets (16.7%), or mL (11.1%) per frog per meal in most facilities. Ad libitum feeding is practiced in two facilities, and two other facilities feed the amount consumed in the period of 30 min (each 11.1%).

In most facilities the amount of feed is weighed, measured, or counted (44.4%), or quantified based on staff experience (44.4%). Further 22.2% determine the amount of feed according to the animals´ behavior. The actual feeding is performed by hand with (88.9%) or without (5.6%) observation of the animals during the feeding process. No facility reported using an automated feeder system.

**Figure 1:**
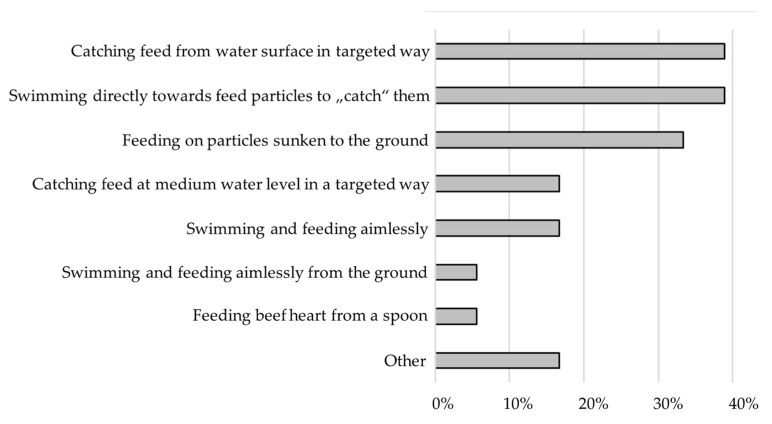
Feeding behavior of *Xenopus laevis* reported in the questionnaire.

The animals usually need less than ten minutes to consume the diet (4–5 min: 22.2%; 6–10 min: 22.2%), or will not consume the total amount (38.9%). Most respondents report a targeted feeding behavior from the water surface (38.9%) or swimming toward the feed particles (38.9%). Another 33.3% report that the frogs feed on particles sunken to the ground or in the mid-water column (16.7%). Non-targeted, aimless feed intake is reported by 16.7% of the respondents (Figure 1).

The six facilities reporting that frogs feed on particles sunken to the ground use sinking diets (5× feed sinking quickly, 1× feed sinking slowly). In four of these facilities, it was reported that there were always feed leftovers. Feed leftovers are not reported for slowly sinking diets (n = 4) and only 3 times with swimming diets (n = 8). The diets sinking to the ground were also associated with relatively high water levels in the tanks (odds ratio of sinking diets fed in a tank 21–50 cm deep: 28.0). The odds ratio of feed leftovers with quickly sinking diets compared to not quickly sinking diets was 6.0, which may indicate that this feeding method may not be ideal for the frogs.

The cleaning intervals of the tanks depend on the amount of dirt (38.9%) or are at a fixed schedule of once (27.8%), twice (11.1%), or three times (11.1%) a week.

### 3.2. Calculations

In five cases, a ration calculation with an amount of feed (g) per animal per week could be conducted. For this, we used the average number of animals per tank and the amount per feed given per animal or per group per meal/week, combined with the number of meals in the respective facility. The N-free extracts (NfE) were calculated as follows: dry matter–crude protein–raw fat–crude fiber–crude ash. Gross energy (GE) was calculated using standard coefficients (23.9 kJ [5.7 kcal]/g crude protein, 39.8 kJ [9.5 kcal]/g crude fat, 20.1 kJ [4.8 kcal]/g crude fiber, 17.5 kJ [4.2 kcal]/g NfE; [15]). Gross energy intake ranged from 73 to 84 kJ per frog per week (mean 75.1, n = 3), protein intake from 1.37 to 5.4 g per frog per week (mean 3.6 g; Table 1). The rations are abbreviated with letters (A: mixture of Tetra Gammarus [dried *Gammarus pulex*], Skretting Optiline ME 1P fish feed and Söll Organix Shrimp Sticks; B: Ssniff Xenopus diet; C: Interquell Fischfit fish feed; D: Nasco PMI CU Adult Frog Diet; E: Skretting Royal Horizon Pellets 2.3).

To calculate the nutrient intake of the abovementioned rations (Table 1) for an individual, an average bodyweight of 70 g was assumed [7]. The intake of GE, DM, CP, CF, RF, Ca, and P was expressed on the basis of metabolic body weight (kg^0.75^ bodyweight) per day in Table 2.

For another eleven cases, the nutrient content of the ration fed could be obtained (based on DM) and compared to amphibian recommendations [9] (Table 3). The GE content of the diets fed ranged from 19.1 to 23.7 kJ/kg DM (mean 21.0 MJ GE; n = 5). Protein was the major source of GE (56.0%), followed by NfE (22.3%) and fat (20.0%). The recommended protein content was met by all rations, except for one with a marginal supply (ration G 43.4% DM, recommendation 44.4% DM). The mean raw fat content in the rations was 14.1 ± 6.3% DM (range 6.8–24% DM, n = 10). For crude fiber, the mean content in the rations was 3.1 ± 1.3% DM (range 1.1–5.7% DM, n = 9); for NfE it was 21.7 ± 12.9% DM (range 2.1–37.1% DM, n = 8). The ration abbreviations are complementary to those from Table 1 and Table 2 (A–E same as above; F: mixture of SDS Aquatic 3, Skretting Royal Horizon Pellets 2.3, and Skretting Elite FR fish feed; G = SDS Aquatic 3 fish feed; H: Bio-Oregon BioVita Fry fish feed; I: Xenopus Express Floating Frog Food 2; J: Xenopus Express Floating & Sinking Frog Food).

The recommended level of calcium and phosphorus was met in all rations with the respective nutrient data given (n = 6 for calcium and n = 8 for phosphorus). In one Xenopus diet and one diet for amphibians and fish, the calcium content was markedly higher than recommended (700% and 417%, respectively). The Xenopus diet with the high calcium content also had a phosphorus content of 822%, the recommended level.

Two diets (B & C in Table 3) had a lower labelled iron and copper content than recommended. One other Xenopus diet had a high iron content of nearly 6 times the recommendation. The zinc content in two diets (C & D in Table 3) was >7 times the recommended level. All diets with information on the vitamin A concentration met or exceeded (5–7.8 times the recommendation; D & G in Table 3) the recommendation.

## 4. Discussion

In comparison to species like rat (*Rattus norvegicus*) and mouse (*Mus musculus domesticus*), *Xenopus laevis* frogs are not used in high numbers as laboratory animals for basic research. In the report about the number of laboratory animals used for experimental purposes under §7, Section 2 and §4, Section 3 of the EU Animal Protection Act in 2020, the percentage of Xenopus frogs is given as 0.57% and 0.19%, respectively, of all animals used in Germany [16]. Even though this is a low percentage, *Xenopus laevis* are used for a wide variety of research purposes, as seen in the responses to the questionnaire. Most frogs are kept in university research facilities, where fundamental research is performed.

The housing systems seem to be quite standardized. Plastic or glass tanks with automated water circulation systems are the most commonly used enclosures. Enrichment mainly consists of some kind of shelter where the frogs have the possibility to move into a cave-like space. In the literature, there are reports that *Xenopus laevis* use enrichment items such as pipes [17] or objects offering refuge like rocks, tiles, and clay pots [18]. It is speculated that such objects enable the frogs to hide from more dominant animals in a group or that they can wait for prey behind such cover material [18]. In the authors´ experience, the shelters are used in case of a sudden increase in light intensity or if the animals seek refuge from a stressful stimulus. However, a correlation between the presence of shelters and growth was not determined [19]. The use of natural materials does not seem feasible in the laboratory setting, especially if the Xenopus frogs are kept in a facility that has to ensure a specified-pathogen-free environment for several species. Complex structures made of enrichment material could hinder health monitoring and removing individual animals from the tank. From all information available, simple, hygienic enrichment items should be used in *Xenopus laevis* tanks to offer the possibility for hiding behavior.

In the questionnaire, the nutritional status of the frogs was described as normal or, in most cases, well-nourished. However, there is no defined body condition score specifically for Xenopus frogs, and scores for other frog species [20] cannot be easily adapted to *Xenopus laevis*. This makes it hard to define the actual condition of the animals. For other amphibians, body condition indices like the scaled mass index or the residual index have been suggested besides the body size indicator snout vent length [21,22]. It is worth further research to find a suitable score or index system to evaluate the body condition of Xenopus species for the sake of animal welfare and experimental documentation and standardization. In other species, the weight gain and/or weight loss combined with body condition scoring is routinely used to evaluate the severity of an experiment. The development of a body condition scoring system for Xenopus frogs would contribute to their health monitoring in terms of the 3R [23], improving animal welfare and experimental results.

The feeding regimen of *Xenopus laevis* shows a high variation between facilities. The frequency of meals ranges from less than once a week to five times a week according to the responses to the questionnaire. In their natural habitat, Xenopus frogs can survive long fasting periods of up to 24 months [10]. Body weight decreases through utilization of fat reserves and, later on, protein catabolism [10]. The availability of prey will naturally be variable from day to day, so that intervals between meals of more than a day should be tolerated well. In the literature, the recommended feeding frequency is once to twice a week [4]. There is evidence that more frequent feeding may accelerate sexual maturation. However, depending on the system used, water quality is better when fewer meals are fed, resulting in a lower workload for feeding and cleaning the tanks [4,7]. In the questionnaire responses, there was no systematic pattern linking feeding and tank cleaning frequency.

Most facilities in the present survey use some kind of commercial diet to feed their *Xenopus laevis* frogs, which is in contrast to earlier reports about Xenopus feeding [10], where more homemade feed items were used. The use of commercial complete diets may mostly stem from logistical reasons. Commercial dry diets are easy to purchase, store, and distribute. More than half of the facilities answering the questionnaire use special Xenopus diets, which are sold by some laboratory animal feed manufacturers, mostly in pelleted form. Fish feed is also commonly used, either alone or in combination with frog diets. Fattening diets for trout often have a similar nutrient content to the frog diets and may be easier to purchase in some countries. A few facilities feed additional items such as beef heart, raw chopped liver, or live earthworms. Raw liver is fed by one facility in case the egg quality declines. The source of liver (e.g., beef, pig or fish) was not specified in the questionnaire response. The addition of liver may be interpreted as a repletion with vitamin A, which is contained in raw liver in high amounts. Vitamin A is important for reproductive success and development in most species [24], including amphibians [25,26]. Another potential benefit of liver as a supplementary feed may be the high protein content. However, the content of protein and vitamin A with the main diet fed in the facility supplementing raw liver (ration D in Table 3) exceeds the recommended levels. Thus, the effect of feeding liver is not clear from the perspective of nutrient supply.

The natural feeding behavior of Xenopus frogs is described as mainly consuming feed items or prey sinking through the water [4,8,9,10,27]. The snatching of prey and shoveling it into the mouth with the forelimbs is characteristic. In this context, it was surprising that nearly half of the diets (44.4%) fed to laboratory Xenopus frogs remained floating on the water surface. Catching feed particles from the water surface in a targeted way was the behavior described most often (38.4%). Thus, the frogs seem to be able to recognize floating feed as “prey” and will start a targeted approach to catch it. Interestingly, frogs may also feed from the ground (33.3%), which is contrary to literature reports [10]. Possibly, the frogs fed sinking diets have learned to feed from the ground to a certain degree. However, it seems to make a difference how fast the feed particles sink for the frogs´ success in “catching” them, because in contrast to floating or slowly sinking diets, the quickly sinking diets were associated with feed residues. Leftover feed will be ignored and not consumed by the frogs [28]. Reports of frogs feeding from the water surface are surprising, given that Ramelow [10] states that Xenopus frogs do not see feed pellets floating on the water surface. The association between quickly sinking diets and leftovers may indicate that this feeding method may not be ideal for the frogs. Specific research on preference of feed characteristics (floating vs. sinking) is necessary, but it seems that swimming and/or slowly sinking feed particles may be better than quickly sinking feed.

In the laboratory setting, the meal size is usually determined by the number of frogs in a tank, as most facilities define the amount of feed in a unit per animal. The usual amount of diet per number of animals is mostly based on staff experience and the behavior of the animals—continuing feeding behavior or restlessness after the initial feeding is interpreted as a sign that more feed is necessary. To a certain degree, this is a feasible method to adjust the amount of diet per tank. Green [7] recommended to feed as much as will be consumed in 15–20 min with only trace leftovers two hours after the meal.

It is hard to determine a general recommendation for the amount of energy and thus diet for a Xenopus frog, since it depends on many factors like age, sex, reproductive state, season, and water temperature [4]. There was no systematic pattern regarding water temperature and feeding frequency in the responses to the questionnaire. However, the approximate GE intake could only be calculated in four cases.

Five responses of the questionnaire allowed calculating a weekly feed intake per animal (Table 1), given the average number of animals per tank in the respective facility. Assuming that the offered feed is consumed without significant leftovers, the DM and energy intake showed high variation (1.44–5.67 g DM and 71.4–88.2 kJ GE per kg^0.75^ bodyweight per day, average 70 g bodyweight assumed [7]). The range of GE intake is in accordance with the energy requirement of reptiles [15], which is much lower than that of homoiothermic animals [29]. Therefore, the calculations in this study can be regarded as plausible. It remains unclear whether the broad range of GE intake is due to variations in husbandry and therefore energy requirement, or whether the supply of feed is too low or too high in some of the facilities. Because of the capacity of many amphibians to overfeed [29], the voluntary intake may not be the ideal marker for an adequate amount of feed. There is also the possibility that the actually consumed amount of feed differs from the average amounts given in the questionnaire, i.e., higher amounts fed in ration A or frogs not consuming the whole amount in rations D and E. There is evidence that feed intake influences digestibility in Xenopus frogs [30], so that the adequate amount of feed will affect the phenotype. Systematic studies are needed to determine the adequate energy supply for Xenopus frogs depending on water temperature and other factors.

The labelled protein content of all rations reported in the questionnaire (Table 3) was at least 98% of the recommended level [11], with protein being the major energy source. The protein/GE ratio was 23.4 ± 1.7 g/MJ. This is in accordance with literature on feeding practice and recommendations for *Xenopus laevis* [4,10,11,31]. Protein utilization is known to be the major energy source in this species [30]. Further research on species-specific nutrient requirements [32] is certainly necessary.

In the cases with labelled information, the supply of minerals, trace elements, and vitamins was quite variable (Table 3). If such levels are tolerated without obvious health problems, the frogs my not be sensitive to high supplies with, e.g., copper [33] or vitamin A as some other species are. It is also possible, however, that some diets have inadequate nutrient levels that result in subclinical states of malnutrition.

## 5. Conclusions

The feeding of *Xenopus laevis* is not standardized across institutions. This is likely to affect research carried out with the frogs. Nutrient intake as well as the type of the diets (sinking vs. floating) should be considered for animal welfare aspects and experiment results. Thus, further research in the needs of *Xenopus laevis* is necessary.

## Figures and Tables

**Table 1:** Rations calculated from the questionnaire responses, compared to recommendations [11] (energy and nutrient intake per week per frog; diet nutrient content as labelled by manufacturers).

Ration		GE [kJ] *	DM [g]	CP [g]	CP/GE Ratio * [g/MJ]	RF [g]	CF [g]	NfE [g] *	Ca [mg]	P [mg]	Ca/P Ratio *	Fe [mg]
		*Intake per Frog per Week*										
A	Dried Gammarus pulex, fish feed, shrimp feed	n.c.	1.37	0.62	n.c.	0.23	0.04	0.30	11.0	4.7	2.3	n.c.
B	Extruded Xenopus diet	68.0	3.21	1.60	23.5	0.27	0.11	1.01	35.6	35.6	1.0	0.27
C	Fish feed for salmonids	73.3	3.51	1.66	22.6	0.29	0.08	1.23	52.1	41.3	1.3	0.28
D	Diet for adult Xenopus	84.0	4.40	2.20	26.2	0.30	0.25	0.90	185.0	110.0	1.7	2.55
E	Fish feed pellets for trout	n.c.	5.40	2.64	n.c.	1.20	n.c.	n.c.	n.c.	66.0	n.c.	n.c.

GE = gross energy, DM = dry matter, CP = crude protein, RF = raw fat, CF = crude fiber, NfE = N-free extracts; Ca = calcium, P = phosphorus, Fe = iron, n.c. = not calculated because of missing nutrient contents; * calculated values.

**Table 2:** Estimated daily nutrient intake of an average 70 g frog fed the ration listed in Table 1.

Ration		GE [kJ] *	DM [g]	CP [g]	RF [g]	CF [g]	NfE [g] *	Ca [mg]	P [mg]
		*Intake per Day per kg^0.75^ Bodyweight of an Average 70 g Frog*							
A	Dried Gammarus pulex, fish feed, shrimp feed	n.c.	1.44	0.65	0.24	0.04	0.31	11.6	4.9
B	Extruded Xenopus diet	71.4	3.37	1.68	0.28	0.12	1.06	37.4	37.4
C	Fish feed for salmonids	77.0	3.68	1.74	0.30	0.08	1.29	54.7	43.4
D	Diet for adult Xenopus	88.2	4.62	2.31	0.31	0.26	0.94	194.2	115.5
E	Fish feed pellets for trout	n.c.	5.67	2.77	1.26	n.c.	n.c.	n.c.	69.3

GE = gross energy, DM = dry matter, CP = crude protein, RF = raw fat, CF = crude fiber, NfE = N-free extracts; Ca = calcium, P = phosphorus, n.c. = not calculated because of missing nutrient contents; * calculated values.

**Table 3:** Labelled energy and nutrient content of Xenopus diets compared to recommendations (gross energy calculated with standard values; selected nutrients).

		GE MJ/kg *	CP %	CP/GE g/MJ *	RF %	CF %	NfE %*	Ca %	P %	Ca/P Ratio *	Fe mg/kg	Cu mg/kg	Zn mg/kg	Vit A IE/kg
		Energy and Nutrient Content on Dry Matter Basis												
**Recommendation** by Ferrie et al. [11]			44.4					0.6	0.3		97.0	12.0	18.0	2914
**Ration**														
A	Dried *Gammarus pulex*, fish feed, shrimp feed	n.c.	45.6	n.c.	17.0	3.1	21.6	0.8	0.3	2.7	n.c.	n.c.	n.c.	n.c.
B	Extruded Xenopus diet	21.2	50.0	23.6	8.3	3.3	31.7	1.1	1.2	0.9	83.3	4.4	20.0	8889
C	Fish feed for salmonids	20.9	47.3	22.6	8.1	2.4	35.2	1.5	1.2	1.3	80.2	11.6	128.9	9626
D	Diet for adult Xenopus	19.1	50.0	26.2	6.8	5.7	20.5	4.2	2.5	1.7	579.5	18.2	130.7	15,438
E	Fish feed pellets for trout	n.c.	48.9	n.c.	22.2	n.c.	n.c.	n.c.	1.2	n.c.	n.c.	n.c.	n.c.	n.c.
F	2 fish feeds, 1 amphibian feed	n.c.	47.0	n.c.	16.5	2.0	19.3	n.c.	1.2	n.c.	n.c.	n.c.	n.c.	n.c.
G	Amphibian diet	20.0	43.3	21.7	7.2	2.6	37.1	2.5	1.3	1.9	194.0	20.2	91.4	22,760
H	Fish feed for salmon and trout	23.7	54.6	23.0	24.0	1.1	6.0	n.c.	n.c.	n.c.	n.c.	n.c.	n.c.	n.c.
I	Floating frog diet	n.c.	45.6	n.c.	13.3	4.4	2.1	1.3	n.c.	n.c.	n.c.	n.c.	n.c.	10,000
J	Floating & sinking frog diet	n.c.	50.0	n.c.	17.8	3.3	n.c.	n.c.	1.3	n.c.	n.c.	n.c.	n.c.	n.c.

GE = gross energy, CP = crude protein, RF = raw fat, CF = crude fiber, NfE = N-free extracts, Ca = calcium, P = phosphorus, Fe = iron, Cu = copper, Zn = zinc, Vit = vitamin; n.c. = not calculated because of missing nutrient contents; * calculated values.

## Data Availability

All data reported in this publication comply with the relevant Ethical Review Board requirements. Original data not given in the manuscript can be made available upon reasonable request to the corresponding author.

## Supplementary Materials

The following are available online at https://www.mdpi.com/article/10.3390/ani12091163/s1, Table S1: Questions asked in the survey with the respective answer options; Table S2: Global distribution of questionnaire responses.
