# Supplementary material for: Current Feeding Practice of Xenopus laevis in a Laboratory Setting
Source: Animals (Basel). 2022 May 1;12(9):1163. doi: 10.3390/ani12091163 (PMC9101390; doi:10.3390/ani12091163)
Supplement: Supplementary file 1 [file animals-12-01163-s001.zip › animals-1699585-supplementary.pdf]

Supplementary material

Table S1. Questions asked in the survey with the respective answer options (18 responses in total).

	Question	Answer options	n	%
1*	Please name your institution. We will not save this information together with the rest of the data and interpret all answers anonymously. This does only serve to identify potential duplicate responses from one facility.	*[free space for text]*	17	94.44
2	Please choose your professional group.	- Animal technician<br>- Veterinarian<br>- Expert vet in lab animal science<br>- Scientist in a different field than veterinary medicine<br>- Lab technician<br>- Biology technician<br>- Other	4<br>1<br>1<br>9<br><br>2<br>0<br>1	22.2<br>5.56<br>5.56<br>50.00<br><br>11.11<br>-<br>5.56
3	Please choose the category of the institution you work for.	- University / institution of public law<br>- Pharmaceutical company with research department<br>- Biotechnology company<br>- Commercial breeder<br>- Commissioned research<br>- Other	17<br>0<br><br>1<br>0<br>0<br>0	94.44<br>-<br><br>5.56<br>-<br>-<br>-
4	How long has your institution been keeping *Xenopus laevis* frogs?	- < 1 year<br>- 1 – 3 years<br>- 4 – 5 years<br>- 6 – 10 years<br>- 11 – 15 years<br>- >15 years	0<br>2<br>0<br>3<br>1<br>12	-<br>11.11<br>.<br>16.67<br>5.56<br>66.67
5	How many adult Xenopus are you currently keeping?	- ≤ 50<br>- 51 – 100<br>- 101 – 300<br>- > 300	3<br>1<br>7<br>7	16.67<br>5.56<br>38.89<br>38.39
6	What kind of husbandry system do you use?	- Removable plastic tanks with automatic water circulation (e.g. Techniplast™)<br>- Permanently installed glass tanks with automatic water circulation (e.g. Aqua Schwarz)<br>- Plastic tanks/tubs without automatic water circulation<br>- Other	10<br><br>4<br><br><br>2<br><br>2	55.56<br><br>22.22<br><br><br>11.11<br><br>11.11
7	How many animals are there in a tank, on average?	- ≤ 5<br>- 6 – 10<br>- 11 – 15<br>- 16 – 25<br>- > 25	3<br>6<br>6<br>2<br>1	16.67<br>33.33<br>33.33<br>11.11<br>5.56
8	At which temperature are you keeping the frogs?	- < 18 °C<br>- 19 – 20 °C<br>- 21 – 22 °C	8<br>9<br>1	44.44<br>50.00<br>5.56

		- > 22 °C	0	-
9	At what water level are you keeping the frogs?	- < 13 cm<br>- 14 – 20 cm<br>- 21 – 30 cm<br>- 31 – 40 cm<br>- 41 – 50 cm<br>- > 50 cm	0<br>9<br>5<br>0<br>2<br>2	-<br>50.00<br>27.78<br>-<br>11.11<br>11.11
10	What kind of enrichment do you use for the frogs?	- Plastic plants<br>- Plastic shelter / house<br>- Real plants<br>- Mangrove roots or similar items<br>- No enrichment<br>- Other (please specify) *[space for text]*	3<br>10<br>0<br>0<br>4<br>6	16.67<br>55.56<br>-<br>-<br>22.22<br>33.33
11	What light cycle is set for the frogs?	- 12 h day / 12 h night<br>- 10 h day / 14 h night<br>- 14 h day / 10 h night<br>- Natural light through window, no artificial light source<br>- Other (please specify) *[space for text]*	15<br>1<br>1<br>0<br><br>1	83.33<br>5.56<br>5.56<br>-<br><br>5.56
12	Please estimate the light intensity parameter in the tanks. (1lx moon light, 25lg underground garage, 100lx living room on a cloudy day, 250lx dining table light, 400lx surgery room or corridor lighting)	*[slide control from 0 to 500 lux]*	18	100
13	Please estimate the nutritional status of your animals.	- Skinny<br>- Quite thin<br>- Normal<br>- Rather well nourished<br>- Obese	0<br>0<br>6<br>12<br>0	-<br>-<br>33.33<br>66.67<br>-
14	How often do you feed the frogs?	- < 1 x weekly<br>- 1 x per week<br>- 2 x per week<br>- 3 x per week<br>- Daily<br>- Different feeding intervals – please specify *[space for text]*	1<br>1<br>4<br>9<br>0<br>3	5.56<br>5.56<br>22.22<br>50.00<br>-<br>16.67
15	How do you feed the animals? *[multiple choice possible]*	- Commercial diet for *Xenopus laevis*<br>- Commercial fish feed<br>- Live feed animals<br>- Beef heart<br>- Home-made diet	10<br>9<br>1<br>2<br>2	55.56<br>50.00<br>5.56<br>11.11<br>11.11
16	What format foes the feed you use have? *[multiple choice possible]*	- Pelleted<br>- Extruded, like dry kibble for dogs<br>- Flakes<br>- Pelletede extrudate<br>- Hybrid pellet<br>- Other (please specify) *[space for text]*	15<br>0<br>0<br>3<br>0<br>1	83.33<br>-<br>-<br>16.67<br>-<br>5.56
17*	In case you use several feed items, which type of feed is the major part of the overall Xenopus feeding?	*[space for text]*	7	38.89

18*	In case you use commercial diets, please name brand and product.	*[space for text]*	15	83.33
19	How does the feed you use move in water?	- It floats<br>- It sinks to the ground quickly<br>- It sinks to the ground slowly	8<br>6<br>4	44.44<br>33.33<br>22.22
20	How do you assign the amount of food per feeding and tank?	- Based on GV SOLAS recommendation<br>- Based on staff experience<br>- Based on the animals´ behavior after the meal<br>- Body weight of the animals / weight development<br>- Appearance of the animals<br>- Other (please specify) *[space for text]*	3<br><br>11<br>7<br><br>2<br><br>1<br>1	16.67<br><br>61.11<br>38.89<br><br>11.11<br><br>5.56<br>5.56
21	Which amount do you feed? Please give an amount per feed item with a suitable reference unit (grams / volume / number per x animals per tank, x times a week)	*[space for text]*	18	100
22	How do you determine the amount of feed to be fed?	- Weighing, measuring / counting<br>- Based on staff experience<br>- According to the animals´ behavior<br>- Automated feeding system<br>- Other (please specify) *[space for text]*	8<br>8<br>4<br>0<br>1	44.44<br>44.44<br>22.22<br>-<br>5.56
23	How do you actually feed the frogs?	- By hand, observing the animals<br>- By hand, without observing the animals<br>- Automated feeder system<br>- Other (please specify) *[space for text]*	16<br>1<br>0<br>1	88.89<br>5.56<br>-<br>5.56
24	How long does it take until the frogs have taken in the complete amount of food?	- 1 – 3 min<br>- 4 – 5 min<br>- 6 – 10 min<br>- > 10 min<br>- The feed will not be consumed completely, there are always leftovers	0<br>4<br>4<br>3<br>7	-<br>22.22<br>22.22<br>16.67<br>38.89
25	How is the feeding behavior of the frogs?	- The frogs “catch” feed from the water surface in a targeted way<br>- The frogs “catch” feed at medium water level in a targeted way<br>- The frogs feed on particles sunken to the ground<br>- The frogs swim around and feed aimlessly<br>- The frogs swim directly towards feed particles to “catch” them<br>- Other behavior (please describe) *[space for text]*	7<br>3<br>6<br>3<br>7<br>5	38.89<br>16.67<br>33.33<br>16.67<br>38.89<br>27.78
26	Do you give supplementary feed or other things in addition to the regular diet?<br>If yes, what exactly and how often?	- Yes<br>- No<br><br>- If yes, what? *[space for text]*	1<br>16<br><br>2	5.56<br>88.89<br><br>11.11

27	How often do you clean the tanks?	- Depending on the feeding intervals<br>- Depending on the amount of dirt<br>- 1x per week, independent of feeding intervals and the actual amount of dirt<br>- 2 x per week, independent of feeding intervals and the actual amount of dirt<br>- 3x per week, or more often	2<br>7<br>5<br><br>2<br><br>2	11.11<br>38.89<br>27.78<br><br>11.11<br><br>11.11
29	For what purpose do you use the frogs?<br>*[multiple choice possible]*	- Spawning for reproductive medicine<br>- Spawning for other research, not reproductive medicine<br>- Organ removal<br>- Toxicity / substance testine<br>- Behavioral research<br>- Other (please specify) *[space for text]*	4<br>14<br><br>6<br>1<br>1<br>5	11.11<br>77.78<br><br>33.33<br>5.56<br>5.56<br>38.89
The question mode was single choice if not stated otherwise.<br>All questions were mandatory, except for those marked with an asterisk (*).				

Table S2. Global distribution of questionnaire responses.

**Region**	**Number of responses**
Europe	10
Northern America	3
Southern America	2
Western Asia	1
No region specified	1
